# Impact of transcutaneous auricular vagus nerve stimulation (taVNS) on cognitive flexibility as a function of task complexity

**DOI:** 10.3389/fnhum.2025.1569472

**Published:** 2025-09-04

**Authors:** Patricio Mena-Chamorro, Tomás Espinoza-Palavicino, Mauricio Barramuño-Medina, Tatiana Romero-Arias, Germán Gálvez-García

**Affiliations:** ^1^Departamento de Psicología, Universidad de La Frontera, Temuco, Chile; ^2^Programa de Kinesiología, Facultad de Ciencias de la Salud, Universidad Autónoma de Chile, Temuco, Chile; ^3^Facultad de Ciencias de la Salud, Universidad Europea de Canarias, La Orotava, Spain; ^4^Departamento de Psicología Básica, Psicobiología y Metodología de las Ciencias del Comportamiento, Facultad de Psicología, Universidad de Salamanca, Campus Ciudad Jardín, Salamanca, Spain

**Keywords:** transcutaneous auricular vagus nerve stimulation, cognitive flexibility, task difficulty, switching, concurrent auditory task

## Abstract

**Purpose:**

This study aimed to evaluate the effect of transcutaneous auricular vagus nerve stimulation (taVNS) on cognitive flexibility under different levels of task complexity. The hypothesis was that taVNS would enhance cognitive flexibility more effectively under demanding task conditions.

**Method:**

A within-subject design was used, involving 24 healthy adults who completed a Dimensional Change Card Sorting task combined with an auditory task of varying difficulty levels (low, medium, high). Participants underwent both active and sham taVNS conditions while performing the tasks. The complexity of the auditory task served to reduce cognitive resources available for the cognitive flexibility task, allowing an assessment of how taVNS modulates cognitive flexibility under different task difficulty conditions.

**Results:**

The results show that switch costs in the Dimensional Change Card Sorting task increase with task difficulty. In addition, active taVNS reduced switch costs significantly in the high complexity condition, while no differences were observed in the low and medium complexity conditions. This indicates that taVNS is particularly effective in conditions of higher cognitive demand.

**Conclusion:**

The findings suggest that taVNS enhances cognitive flexibility, especially in more complex tasks, providing a better understanding of the effects of taVNS on cognitive control.

## 1 Introduction

Executive functions are top-down cognitive processes that enable goal-directed behavior, with inhibitory control and cognitive flexibility (CF) as key components. Inhibitory control suppresses dominant responses through attention and behavior regulation, which can be categorized into selective attention and response inhibition (Diamond, 2013). CF refers to the ability to switch between tasks or mental sets rapidly and includes task-switching (changing tasks with different instructions and stimuli) and set-shifting (shifting attention within the same stimuli to follow specific instructions) (Dajani and Uddin, 2015; Diamond, 2013). These functions are fundamental for higher-order cognitive abilities (Diamond, 2013; Miyake and Friedman, 2012).

In this regard, various studies have investigated how CF could be improved through cognitive training (Karbach and Kray, 2009), physical activity (Bherer et al., 2013), and non-invasive brain stimulation techniques, with recent emphasis on transcutaneous auricular vagus nerve stimulation (taVNS) due to its ease of use and implementation, low cost, and lack of adverse effects, except for mild itching (Dietrich et al., 2008; Kraus et al., 2013; Redgrave et al., 2018).

taVNS modulates vagus nerve activity through its left atrial branch (Capone et al., 2015; Farmer et al., 2021; Frangos et al., 2015; Frangos and Komisaruk, 2017). This stimulation generates action potentials in the nerve cells, sending signals to the brainstem, including the nucleus tractus solitarius and locus coeruleus, which is the primary source of norepinephrine in the brain (Sclocco et al., 2019). This process boosts the release of norepinephrine and GABA (Keute et al., 2018; Yakunina et al., 2017). As a result, taVNS enhances brain activity in areas associated with cognitive flexibility (CF), including the anterior cingulate cortex, prefrontal cortex, dorsolateral frontal cortex, motor cortex, and premotor cortex (Fisher and Velasco, 2014; Zaehle and Krauel, 2021). Consequently, it is reasonable to expect that taVNS could improve CF.

However, research specifically examining the effects of taVNS on CF is limited and has yielded mixed results. On the one hand, two studies have found that taVNS improves CF. Borges et al. (2020) assessed executive functions using various tasks (e.g., the Flanker task, Spatial Stroop task, Number-Letter task, and Dimensional Change Card Sorting task). They found that taVNS improved CF, as measured by the Dimensional Change Card Sorting task. No such improvement was observed with other tasks, such as the Spatial Stroop task, which involves different inhibitory processes. Similarly, Driskill et al. (2022) provided evidence from an animal model where invasive vagus nerve stimulation in naïve rats before a CF task improved performance. This aligns with findings suggesting that vagus nerve stimulation can modulate CF-related cognitive processes. On the other hand, Tona et al. (2020) found that taVNS did not improve CF in a switching paradigm in which participants repeatedly switched between high/low or odd/even digit classifications based on previous cues.

It is important to highlight that the previous studies are difficult to compare due to their use of different cognitive paradigms, varying dependent variables, dissimilar stimulation intensities, and intra- and inter-subject designs. These factors complicate a thorough, evidence-based investigation of how taVNS and CF interact. In line with this, it has been suggested (though not directly examined) that taVNS could be particularly beneficial in high-demand scenarios where cognitive processes like conflict resolution in CF require more mental effort (Borges et al., 2020; Chen et al., 2022; Fischer et al., 2018). Therefore, this study seeks to assess the impact of taVNS on CF, focusing on task complexity. A secondary auditory task was introduced in which participants performed a Dimensional Change Card Sorting task concurrently with an auditory task with three levels of task difficulty, in which a verbal response was required. According to central resources/capacity theories (e.g., Gopher, 1986; Kahneman, 1973), performance is impaired when the resources required to complete an ongoing task exceed the available resources. A good way to reduce the availability of resources is to introduce a concurrent task, incite participants to allocate some resources to it, and, therefore, withdraw resources from the primary task. It is hypothesized that taVNS will have its most significant effect in the most challenging task conditions.

## 2 Materials and methods

### 2.1 Participants

This study involved 24 healthy adults (mean age = 23.17 ± 2.76, with twelve women) selected through non-probability intentional sampling. A sample size of 22 participants was determined by *a priori* power analysis (*f* = 0.25, power = 0.95, *α* = 0.05; F test family, repeated measures ANOVA; G*Power version 3.1; Faul et al., 2009). This effect size was selected based on Cohen’s (1988) conventions. It was considered appropriate given the within-subjects design and the effect sizes reported in previous taVNS studies using similar paradigms (e.g., Borges et al., 2020). However, two more volunteers were recruited as the experimental conditions were counterbalanced to account for immediate carryover/adaptation effects. Accordingly, a Latin square design was used to counterbalance the order of the twelve experimental conditions (Kim and Stein, 2009). This method’s benefit is that it counterbalances ordinal positions and immediate sequential effects. The design produced twenty-four counterbalanced sequences with twelve experimental conditions. All participants reported normal vision and right-handedness as determined by the *“Edinburgh Handedness Inventory”* (Albayay et al., 2019). None of the participants had prior experience with taVNS or had taken part in previous taVNS studies. Exclusion criteria were established based on prior research on CF and taVNS (e.g., Borges et al., 2020) and excluded individuals with psychiatric or neurological disorders, consumption of drugs, alcohol, or coffee within 24 h of the experiment, a history of brain surgeries, tumors, intracranial implants, or musculoskeletal injuries of the upper or lower limbs. Participants were informed about anonymity (ensured through identification), confidentiality, and their rights, and written informed consent was obtained before participation. They were also informed that they could withdraw from the study without any adverse consequences. After completing the experiment, participants were fully briefed on the study’s purpose. The study protocol received approval from the Research Ethics Committee of Universidad de La Frontera (No. 081/23) and adhered to the principles of the Helsinki Declaration.

### 2.2 Tasks, apparatus and stimuli

#### 2.2.1 Cognitive flexibility task

The Dimensional Change Card Sorting task, based on Hindy and Spivey (2008), measured CF through switch cost. Next, basic concepts about switch costs will be introduced. A task set refers to a specific group of processes organized to perform a particular task. When these processes need to be reorganized to accommodate a new task or situation, this is known as a “set switch.” Compared to situations where no task changes, switching tasks typically results in temporary performance declines, which can be reflected in reduced accuracy and longer reaction times. This phenomenon is referred to as the “*switch cost*.” The most common explanation for this effect is that it requires a participant to undergo a “*task set shift*,” which includes shifting attention from one task to another. This shift involves two key components: activating the new task (i.e., disengaging from the first task, switching, and re-engaging with the new task) and inhibiting the previous task set (Hübner and Druey, 2006; Koch et al., 2010). Importantly, the literature generally agrees that the switch cost is a reliable indicator of CF (Diamond, 2013).

**Figure 1:**
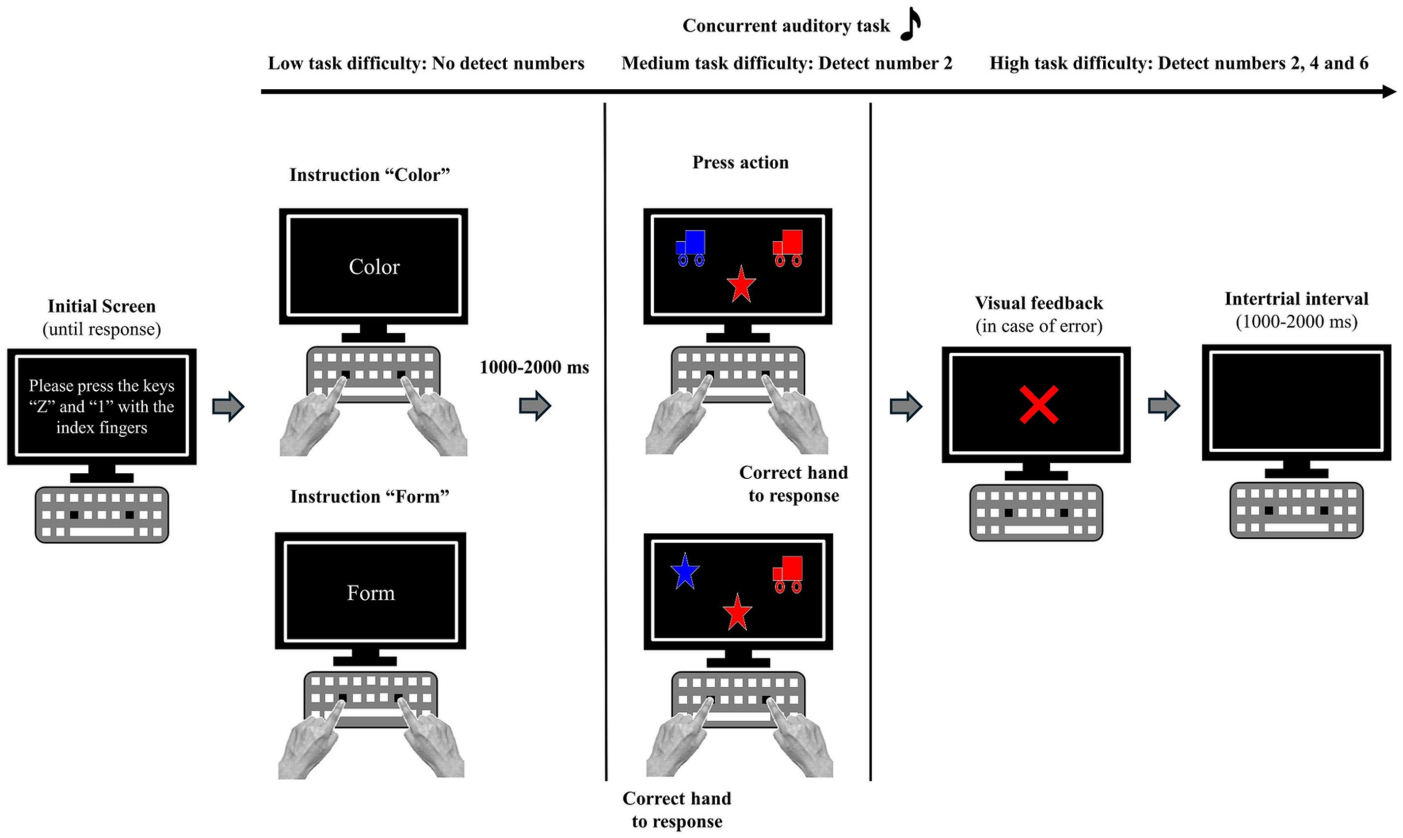
Schematic drawing of the CF and auditory tasks sequence.

The task began with a screen instructing participants to place their index fingers on the “Z” key (left index finger) and the “1” key (right index finger) on the keyboard. Then, a screen appeared with the task instructions, displaying the words *“shape”* or *“color”* in the center of the screen (i.e., respond to the target stimulus presented at the top of the screen that matched either the color or the shape). The target stimuli appeared after a random interval between 1,000 and 2000 ms in the upper-left and upper-right corners of the screen, with a visual separation of approximately 16 degrees. The color and shape of the stimuli were different, with the target stimulus matching only one feature (either color or shape). Participants were required to consider the target stimulus corresponding to the classification dimension and press the key ipsilateral to the target stimulus. Visual feedback was provided after the error trials through a bitmap image with a red “X.” Finally, a random interval between 1,000 ms and 2000 ms was used between each trial to reduce the tendency for rhythmic responses (see Figure 1 for a visual description of the task procedure). The classification rule changed every two trials (i.e., AABB) to obtain no-switching and switching trials in each experimental block. The difference in performance between no-switch and switch trials reveals the switch cost. It is important to highlight that all stimuli were presented with the same frequency to control for potential biases related to the stimulus and/or its location and the corresponding response.

The Dimensional Change Card Sorting task involved a standard QWERTZ keyboard and a 19-inch monitor on which images were presented at a temporal sampling rate of 100 Hz. Participants sat approximately 60 cm from the screen to perform the task. Four bitmap images were included as classification objects: a blue truck, a red truck, a blue star, and a red star. Each image measured 5 cm by 4.9 cm (200 × 190 pixels). The task was designed using Open-Sesame software.

#### 2.2.2 Auditory task

The auditory task was inspired by Michael et al. (2014). Subjects had to complete an auditory task concurrently with the visuomotor task; the difficulty of the tasks varied (see Figure 1). They had to either (a) listen to the auditory list but not respond to it, (b) detect a single target (i.e., the number *“two”*) by emitting a verbal response (i.e., say the word “top”) when they heard that target, or (c) detect one of three possible targets (i.e., the numbers *“two,” “four”* and *“six”*) by emitting the same type of verbal response. These three blocks resulted in a task of low, medium, and high complexity (i.e., detecting zero, one, and three items, respectively). The target(s) appeared randomly and unpredictably. The three degrees of difficulty were presented within three separate blocks, during which each subject performed a constant visual task (i.e., the CF task remained constant since the aim was to assess the effects of a secondary task on CF). To optimize time-sharing between the visual and auditory tasks (Wickens et al., 1984), the auditory items were played continuously from the beginning of the block until the end, without interruption. Furthermore, the high throughput of auditory items (1 per second) was introduced to make the task resource-demanding, as research has shown that presenting one stream of stimuli at a high rate precludes efficient handling of another stream simultaneously. Therefore, the method we used was a straightforward means of withdrawing resources from the CF task, which allowed us to observe the effects of reduced resource availability. The subjects were informed that the two tasks (i.e., CF and auditory) were equally important and that one should not be favored over the other. Their responses were recorded by the experimenter, who was seated at some distance from each subject and had a complete list of auditory stimuli to refer to.

In this auditory task, the stimuli were the spoken numbers 0 through 9, recorded as an mp3 file and played back with speakers at a rate of 1 per second. The stimuli were arranged in a series of 10 numbers, with all numbers presented randomly and once only. Each new series started only after the previous one had ended, but there was no signal in the mp3 file of the end of one series and the beginning of the next. This arrangement prevented subjects from developing detection and response strategies.

#### 2.2.3 Transcutaneous auricular vagus nerve stimulation

Transcutaneous auricular vagus nerve stimulation (taVNS) was administered using the NEMOS® (Cerbomed, Erlangen, Germany) neurostimulation device, with two titanium electrodes on the left ear. The protocol for optimal taVNS, as outlined by Farmer et al. (2021), was strictly followed: (a) continuous stimulation, (b) pulse width of 200–300 ms at a frequency of 25 Hz, and (c) a 10-s stimulation period followed by a 10-s pause. There were two conditions: an active taVNS condition and a sham taVNS condition, the latter designed to control any placebo effects induced by electrical stimulation (Farmer et al., 2021). In the active taVNS condition, the electrodes were placed on the cymba conchae of the left ear, while in the sham condition, the electrodes were positioned on the left earlobe, an area devoid of vagal fibers (Fallgatter et al., 2003; Peuker and Filler, 2002). As a result, the sham condition did not activate the brainstem or cerebral cortex (Kraus et al., 2013). The stimulation intensity was determined based on each participant’s detection threshold (for further details, refer to Fischer et al., 2018). In both conditions, stimulation was adjusted to be perceptible to the participant without being painful. The average stimulation intensity applied was 1.5 mA (ranging from 0.6 to 3.0 mA) for the active taVNS condition and 1.7 mA (ranging from 0.6 to 3.0 mA) for the sham taVNS condition. There was no difference in the stimulation intensity between both conditions (t_(23)_ = −0.985, *p* = 0.335). The left ear was selected for stimulation because its atrial branch does not have direct efferent fibers projecting to the heart (Butt et al., 2020; Nemeroff et al., 2006), ensuring no abnormal cardiac activity (e.g., Kreuzer et al., 2012).

### 2.3 Procedure

A sham-controlled, single-blinded, within-subject design was used. The Dimensional Change Card Sorting task was administered six times according to the type of block and stimulation (i.e., the different experimental conditions: low complexity—active taVNS, medium complexity—active taVNS, high complexity—active taVNS, low complexity—sham taVNS, medium complexity—sham taVNS, and high complexity—sham taVNS). Each block consisted of 99 trials (594 trials in total) plus 16 practice trials before each block. Due to the large number of trials, the experiment was divided into two sessions (each including three experimental conditions), conducted on two consecutive days at the same time. Each session lasted approximately 60 min.

Before each experimental session, the taVNS electrodes were placed on the cymba conchae of the left ear, and the stimulation intensity was adjusted based on each participant’s detection threshold. Once the intensity was determined, stimulation was delivered continuously throughout the experimental CF and auditory task. After completing the tasks, stimulation was discontinued, and the taVNS electrode was removed. The session concluded with a questionnaire to assess potential side effects of taVNS, following the protocol of Fischer et al. (2018). Participants rated the severity of various symptoms on a scale from one to seven, ranging from minimal to maximum. These symptoms included headache, nausea, dizziness, neck pain, muscle contractions in the neck, stinging sensations under the electrodes, skin irritation in the ear, fluctuations in concentration or emotions, and general discomfort.

### 2.4 Data analyses

For the CF task, the dependent variables analyzed were the reaction times (RT) and error rate (ER). Additionally, the percentages of hits (i.e., correctly detected targets) and false positives were recorded in the auditory task. The independent variables manipulated were task complexity (low vs. medium vs. high), stimulation condition (sham taVNS vs. active taVNS), and trial type (no-switching vs. switching).

Data analysis was conducted in Rstudio (RStudio Team, 2022). For RT and ER in the CF task, factorial repeated measures ANOVA were performed using the “*afex*” package (Singmann et al., 2024). Given the within-subject design, all participants completed each combination of experimental conditions. The within-subjects factors included task complexity (low vs. medium vs. high), stimulation condition (sham taVNS vs. active taVNS), and trial type (no-switching vs. switching). When the assumption of sphericity was violated, Greenhouse–Geisser corrections were applied. Partial eta square (η^2^_p_) was reported as a measure of effect size. Planned comparisons were conducted with Bonferroni correction for *p*-values adjustment using the “*emmeans*” package (Lenth, 2016). For hits and false positives in the auditory task, paired t-test were performed between task complexity conditions, as well as subjective ratings of side effects between active taVNS condition and sham taVNS condition.

## 3 Results

The following data were excluded from the RT analysis: ER (10.23%) and trials with reaction times less than 100 milliseconds (ms) or greater than 1,500 ms (0.72%).

### 3.1 Subjective ratings of side effects

Subjective ratings showed that side effects were minor. However, there was a significant statistical difference between the sham taVNS condition (*M* = 1.76, SD = 0.70) compared to the active taVNS condition (*M* = 2.05, SD = 0.65, *t*_(23)_ = 2.307, *p* = 0.030), with higher ratings in the active taVNS condition. Further analysis of individual side effects using paired t-tests showed no significant differences between stimulation conditions for most side effects, except for the physical subjective experience of the stimulation, with higher ratings for the taVNS condition in stinging sensation, irritation ear, and unpleasant feeling (see Table 1).

**Table 1:** Mean subjective ratings (standard error) for the stimulation side effects in the active and sham condition.

Side effects	Active taVNS	Sham taVNS	t-value	*p*-value
Headache	1.34 (0.78)	1.33 (0.75)	0.000	1.000
Nauseas	1.08 (0.45)	1.12 (0.44)	−0.810	0.425
Dizziness	1.30 (0.75)	1.27 (0.93)	0.093	0.926
Neck pain	1.25 (0.60)	1.40 (0.76)	−1.319	0.199
Stinging sensation	2.73 (1.54)	2.02 (1.09)	2.482	0.020*
Irritation ear	2.44 (1.44)	1.44 (0.79)	4.652	< 0.001***
Fluctuation concentration	3.35 (1.13)	3.15 (1.29)	0.764	0.452
Fluctuation feeling	2.67 (1.22)	2.49 (1.33)	1.007	0.324
Unpleasant feeling	2.23 (1.21)	1.67 (1.25)	2.776	0.010*

*p < 0.050, **p < 0.010, ***p < 0.001.

### 3.2 Auditory task

The percentages of hits (correctly detected targets) and the percentages of false positives observed in the medium and high task difficulty conditions were subjected to paired-sample t-tests. As expected, the percentage of correct detections was higher in the medium difficulty task condition (*M* = 98.3%) than in the high difficulty task condition (*M* = 95.1%; *t*_(23)_ = 4.82, *p* < 0.001). The percentage of false positives was lower in the medium difficulty task condition (*M* = 0.02%) than in the high difficulty task condition (*M* = 0.12%; *t*_(23)_ = 2.29, *p* = 0.031). A signal detection analysis showed that the sensitivity index (d′) was significantly higher in the medium difficulty task condition (*M* = 6.92) than in the high difficulty task condition (*M* = 5.45; *t*_(23)_ = 4.23, *p* < 0.001).

### 3.3 Reaction time (RT)

The repeated measure ANOVA (task complexity × stimulation × trial type) for RT showed that all the main effects were statistically significant. The main effect of task complexity (*F*_(1.02, 23.43)_ = 59.33, *p* < 0.001, η^2^_p_ = 0.721) showed faster RTs for the low task complexity condition (*M* = 554.77 ms, SD = 79.08) compared to the medium task complexity condition (*M* = 593.66 ms, SD = 79.47, *p* < 0.001) and the high task complexity condition (*M* = 649.75 ms, SD = 86.63, *p* < 0.001), with significant differences between the medium task complexity and high task complexity conditions (*p* < 0.001). The main effect of stimulation (*F*_(1, 23)_ = 19.66, *p* < 0.001, η^2^_p_ = 0.461) indicated faster RTs in the active taVNS condition (*M* = 585.09 ms, SD = 88.41) compared to the sham taVNS condition (*M* = 613.70 ms, SD = 90.38). The main effect of trial-type (*F*_(1, 23)_ = 24.63, *p* < 0.001, η^2^_p_ = 0.517) showed faster reaction times for the no switching condition (*M* = 586.10 ms, SD = 87.96) compared to the switching condition (*M* = 612.69 ms, SD = 91.12) (i.e., switch cost).

The first-order interaction task complexity × stimulation was not significant (*F*_(1.34, 30.80)_ = 0.73, *p* = 0.486, η^2^_p_ = 0.031). The interaction of task complexity × trial-type (*F*_(1.27, 29.21)_ = 7.11, *p* = 0.020, η^2^_p_ = 0.236) was significant. Planned comparisons indicated that the switch costs increase with task complexity, with differences observed between the switch cost of the low task complexity condition (*M* = 19 ms) compared to the medium task complexity condition (*M* = 24 ms, *p* = 0.038) and the high task complexity condition (37 ms, *p* = 0.005), with also significant differences between the medium and high task complexity conditions (*p* = 0.035). The interaction of stimulation × trial-type was also significant (*F*_(1, 23)_ = 73.99, *p* < 0.001, η^2^_p_ = 0.763), with a higher switch cost for sham taVNS (*M* = 39 ms) compared to active taVNS (*M* = 15 ms, *p* < 0.001). Finally, the second-order interaction of task complexity × stimulation × trial-type was not significant (*F*_(1.22, 28.15)_ = 1.70, *p* = 0.194, η^2^_p_ = 0.069). However, interesting results and interactions emerged from the planned comparisons.

**Figure 2:**
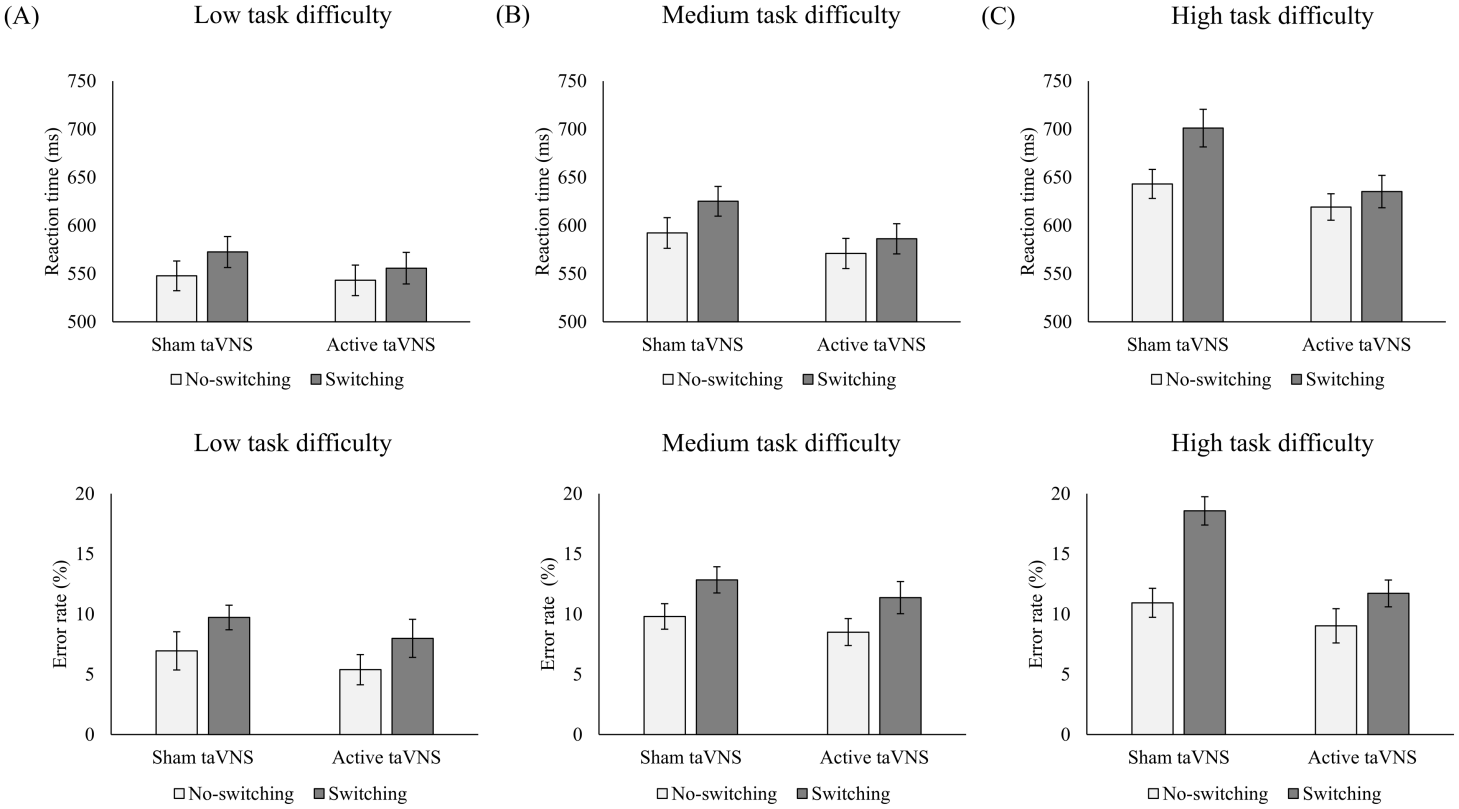
*Upper panel:* Stimulation × trial type interactions within the **(A)** low, **(B)** medium, and **(C)** high task difficulty conditions, with respect to mean reaction time. *Lower panel:* Corresponding interactions for mean error rate. Error bars represent the standard error of the mean.

First, planned comparisons showed no differences for the interaction of stimulation × trial type between the low task complexity and the medium task complexity conditions (*p* = 0.813). However, there was a statistical difference between the low and high task complexity conditions (*p* = 0.015), without differences between the medium and high task complexity conditions (*p* = 0.126). Therefore, the differences in the interaction of stimulation × trial type based on task complexity were located between low and high task difficulty conditions. In this vein, if each task complexity condition is analyzed separately, different patterns of results emerge (see the upper panel of Figure 2). For the low task difficulty condition, although there was a lower switch cost for active taVNS (*M* = 13 ms) compared to sham taVNS (*M* = 25 ms), no statistically significant difference was found (*p* = 0.282). A similar pattern occurs for medium task difficulty, with a lower switch cost for active taVNS (*M* = 15 ms) compared to sham taVNS (*M* = 33 ms), with no statistically significant differences between them (*p* = 0.171). In contrast, for the high task complexity condition, a statistically significant difference was found between the switch cost of the sham taVNS condition (58 ms) compared to a lower switch cost for the active taVNS condition (*M* = 16 ms, *p* < 0.001).

In addition, when comparing the switch costs across the task complexity conditions, differentiating between sham and active taVNS was found. On the one hand, for sham taVNS stimulation, there are no differences between the switch costs of the low task complexity condition (*M* = 25 ms) and the medium task complexity condition (*M* = 33 ms, *p* = 0.444). However, there are differences between the low task complexity condition (25 ms) and the high task complexity condition (*M* = 58 ms, *p* < 0.001), as well as between the medium and high task complexity conditions (58 ms, *p* = 0.013). On the other hand, for active taVNS, the switch costs remain stable, with no differences between them (*M* = 13 ms, *M* = 15 ms, and *M* = 16 ms for the low, medium, and high task complexity conditions, respectively; *p* > 0.600 for all).

### 3.4 Error rate (ER)

For ER (see the lower panel of Figure 2) (repeated measure ANOVA task complexity × stimulation × trial-type), the main effect of task complexity (*F*_(1.55, 35.64)_ = 14.09, *p* < 0.001, η^2^_p_ = 0.380) showed less ER for the low task complexity condition (*M* = 7.51%, SD = 6.98) compared to the medium task complexity condition (*M* = 10.63%, SD = 5.92, *p* = 0.010) and the high task complexity condition (*M* = 12.56%, SD = 7.07, *p* < 0.001), without differences between the medium task complexity and high task complexity conditions (*p* = 0.074). The main effect of stimulation (*F*_(1, 23)_ = 8.00, *p* = 0.010, η^2^_p_ = 0.258) indicated less ER in the active taVNS condition (*M* = 9.00%, SD = 6.43) compared to the sham taVNS condition (*M* = 11.47%, SD = 7.29). The main effect of trial-type (*F*_(1, 23)_ = 26.37, *p* < 0.001, η^2^_p_ = 0.534) showed less ER for the no switching condition (*M* = 8.43%, SD = 6.20) compared to the switching condition (*M* = 12.04%, SD = 7.26, *p* < 0.001) (i.e., switch cost).

For the first-order interactions, the interactions task complexity × stimulation (*F*_(1.72, 39.59)_ = 1.33, *p* = 0.273, η^2^_p_ = 0.055), and stimulation × trial type (*F*_(1, 23)_ = 1.87, *p* = 0.184, η^2^*_p_* = 0.075) were not statistically significant. However, the interaction of task complexity × trial-type was significant (*F*_(1.96, 44.99)_ = 3.34, *p* = 0.045, η^2^_p_ = 0.127). Planned comparisons indicated that although the switch costs did not increase between the low task complexity condition (*M* = 2.69%) compared to the medium task complexity condition (*M* = 2.95%, *p* = 0.819), there is such an increase between the low task complexity condition and the high task complexity condition (*M* = 5.16%, *p* = 0.021), with also significant differences between the medium and high task complexity conditions (*p* = 0.042). Finally, the second-order interaction of task complexity × stimulation × trial-type was not significant (*F*_(1.57, 36.03)_ = 1.81, *p* = 0.174, η^2^_p_ = 0.073). However, interesting results and interactions emerged from the planned comparisons.

First, planned comparisons showed no differences for the interaction of stimulation × trial type between the low task complexity condition and the medium task complexity condition (*p* = 0.990). However, there was a marginal difference between low and high task complexity conditions (*p* = 0.061), and also a marginal difference between medium and high task complexity conditions (*p* = 0.075). In this vein, if each task difficulty condition is analyzed separately, different patterns of results emerge (see the lower panel of Figure 2). For the low task difficulty condition, there was no difference between the switch costs for sham taVNS (*M* = 2.77%) and active taVNS (*M* = 2.60%, *p* = 0.945). Similarly, there was no difference between the switch costs for sham taVNS (*M* = 3.04%) and active taVNS (*M* = 2.86%, *p* = 0.935) for the medium task difficulty condition. In contrast, for high task difficulty, a statistically significant difference was found between the switch cost of the sham taVNS condition (7.63%) compared to a lower switch cost for the active taVNS condition (*M* = 2.69%, *p* = 0.006).

In addition, when comparing the switch costs across the task complexity conditions, differentiating between sham and active taVNS was found. On the one hand, for sham taVNS stimulation, although there were no differences between the switch costs of the low task complexity condition (*M* = 2.77%) and the medium task complexity condition (*M* = 3.03%, *p* = 0.886), significant differences were observed between the low and high (*M* = 7.63%) task complexity conditions (*p* < 0.001), as well as between the medium and high task complexity conditions (*p* = 0.015). On the other hand, for active taVNS stimulation, the switch costs remained stable, with no significant differences between them (*M* = 2.60%, *M* = 2.86%, and *M* = 2.69% for the low, medium, and high task complexity conditions, respectively; *p* > 0.600 for all).

## 4 Discussion

This study aimed to assess the effectiveness of taVNS on CF as a function of task complexity. To this end, two conditions (sham taVNS and active taVNS) were compared in a Dimensional Change Card Sorting task with three levels of task complexity (low, medium, and high), depending on a concurrent auditory task (no number detection, detecting a single number, and detecting three numbers). It was hypothesized that taVNS would be more effective under higher-demand task conditions.

The concurrent auditory task resulted in fewer hits and an increase in false alarms as the complexity of the task increased, confirming that it reduces the availability of cognitive resources by drawing resources away from the primary task (i.e., the CF task). This progressive increase in task complexity led to increased switch costs across the complexity conditions. In this regard, it should be highlighted that the cost of switching between tasks tends to be higher in more complex tasks due to several factors that increase cognitive demands (e.g., task-set reconfiguration, increased interference, cognitive load, etc.; see Kiesel et al., 2010 for more information). However, and in line with our hypothesis, this increase was not uniform across all complexity conditions, depending on the type of stimulation applied. While there were no differences between sham taVNS and active taVNS in the low and medium task difficulty conditions, significant differences were found in the high task difficulty condition. This suggests that active taVNS is especially effective when the task is more complicated, supporting our initial hypothesis. Additionally, as previously mentioned, switch costs increase with task complexity (especially in the most difficult condition) when taVNS is not applied (i.e., sham taVNS). Conversely, switch costs in active taVNS remain stable as task complexity increases. These findings suggest that taVNS can effectively enhance CF, especially under complex conditions, shedding light on its potential for improving executive functions in high-demand cognitive tasks.

Our results confirm the previous findings of Borges et al. (2020) regarding the effects of taVNS on CF. However, these authors found no effect of taVNS on inhibitory control even when using specific paradigms designed to target this function (e.g., the Spatial Stroop task), a finding consistent with previous studies (e.g., Pihlaja et al., 2020; Ventura-Bort et al., 2018). They, therefore, concluded that tasks were not cognitively demanding enough to evoke a decrease in cardiac vagal activity. It should be noted, however, that other studies have reported improvements in inhibitory control with taVNS but only when working memory demands are involved. For example, Beste et al. (2016) reported enhanced response inhibition when working memory was engaged. This may help explain why studies on CF have found effects of taVNS compared to studies focusing specifically on inhibitory control. CF may benefit from taVNS since it improves two subcomponents: inhibitory control and working memory. In this regard, research on working memory has shown that taVNS enhances working memory performance (Burger et al., 2016; Jacobs et al., 2015), possibly due to greater norepinephrine release from the locus coeruleus to the hippocampus (Bahtiyar et al., 2020; Jacobs et al., 2015) and an increase in arousal and attention, which are crucial for memory processes (Burger et al., 2016; Raedt et al., 2011; Roosevelt et al., 2006). Within this framework, Borges et al. (2020) propose that the improvement of CF through taVNS may be mediated by its impact on neurotransmission. Specifically, taVNS stimulates the locus coeruleus (Dietrich et al., 2008), the brain’s primary source of norepinephrine (Foote et al., 1983). Research indicates that norepinephrine plays a key role in the effects of taVNS (e.g., Badran et al., 2018). Furthermore, the locus coeruleus is crucial for reorienting attention and enhancing CF, with its neurons demonstrating task-related activation (Sara, 2015).

Regarding the side effects, it is worth noting that although the subjective effects of stimulation were measured, the differences between the active and sham stimulation conditions were minimal, with a few exceptions (such as itching or ear irritation). These findings suggest that participants only notice physical differences between the sham and taVNS conditions, which is somewhat anticipated given the stimulation intensity applied, consistent with prior studies (Fischer et al., 2018).

Regarding the limitations of this study, it should be highlighted that it implemented the Dimensional Change Card Sort task, utilizing an AABB trial structure instead of the block-based structure used in previous research (e.g., Borges et al., 2020; Yang et al., 2017). Borges et al. (2020) employed a sequential block design where participants first completed a whole block of trials using one sorting dimension (A), followed by a block with the alternate dimension (B), and later a mixed block. However, our study presented trials in an alternating pattern within each block (i.e., two consecutive trials following one sorting rule, then two trials following the other: AABB). This trial-wise alternation allowed us to assess set-shifting more dynamically, capturing both immediate switch costs and short-term adaptation within a continuous sequence. In contrast, the block-based design (e.g., Borges et al., 2020) primarily evaluates the cost of transitioning between sustained task sets, potentially overlooking finer fluctuations in CF. In this manner, our results suggest that the AABB structure provides a more granular measure of switching efficiency, offering insights into how participants adjust to shifting rules in real time. In this vein, future studies should explore how task structure influences performance and whether trial-wise alternation better captures individual differences in CF. In addition, although a clear pattern of benefit from taVNS in CF has been observed, the question remains whether taVNS influences other cognitive processes underlying CF. In this line, Sommer et al. (2023) found that taVNS increased integrative processing in multitasking, where CF is essential, but only in the first block. Consistently, taVNS has also been shown to enhance performance in complex multitasking scenarios such as simulated driving (Espinoza-Palavicino et al., 2023; Gálvez-García et al., 2024), suggesting that similar underlying mechanisms, such as increased arousal and attentional reorientation, may be supporting CF improvements observed in our study. Finally, to complement the understanding of the effect of active taVNS on CF, future studies should include psychophysiological measures to corroborate the proper functioning of taVNS. Although this area is still emerging, and psychophysiological findings are mixed (Borges et al., 2020; Fischer et al., 2018; Ventura-Bort et al., 2018), especially concerning cardiac vagal activity (Borges et al., 2020; De Couck et al., 2017; Wolf et al., 2021), the incorporation of these measures would be critical to deepening the understanding of the underlying mechanisms of taVNS. In this regard, future research could also benefit from considering insights from the heart rate variability (HRV) biofeedback literature, which suggests that enhancing cardiac vagal control, even in a single session, may improve attentional performance, particularly in individuals with high baseline stress (Blaser et al., 2023). Although our study did not measure baseline stress or HRV levels, it is plausible that such individual state factors influence the effectiveness of taVNS. Identifying these potential moderators could help clarify the specific conditions under which taVNS yields the greatest cognitive benefits.

In summary, this study demonstrates how CF is influenced by taVNS, which is in line with scarce previous research. More importantly, it shows how taVNS significantly impacts CF in complex tasks, providing a better understanding of the effects of taVNS on cognitive control.

## Data Availability

The raw data supporting the conclusions of this article will be made available by the authors, without undue reservation.
